# Dynamic changes in ROS-antioxidant-metabolite network in dragon fruit infected with *Neoscytalidium dimidiatum*

**DOI:** 10.1016/j.fochx.2025.102957

**Published:** 2025-08-30

**Authors:** Lili Xie, Xueli Wang, Xiao Ma, Kaiwei Shen, Xuyi Zhang, Xiujun Wang

**Affiliations:** a Guizhou Key Laboratory of New Quality Processing and Storage of Ecological Specialty Food, College of Liquor And Food Engineering, Guizhou University, Guiyang 550025, PR China; b College of Liquor and Food Engineering, Guizhou University, Guiyang 550025, China; c College of Resources and Environmental Engineering, Guizhou University, Guiyang 550025, China

**Keywords:** Dragon fruit canker disease, ROS scavenging enzyme, Primary metabolites, Volatile metabolites, Plant-pathogen interaction, Postharvest defense

## Abstract

This study investigates the effects of *Neoscytalidium dimidiatum* (*N. dimidiatum*) infection on the postharvest quality of dragon fruit. Infection accelerated fruit softening, decay, and weight loss, while increasing the respiration rate and total acid (TA) content, and decreasing soluble solids (TSS) content. Pathogen infection triggered a rapid reactive oxygen species (ROS) burst, leading to oxidative stress, with O_2_^−^ and malondialdehyde (MDA) levels 2.11 and 1.58 fold higher than those of the controls, respectively. Antioxidant enzymes (SOD, CAT, POD, APX) were initially activated (0–2 days) but declined sharply later (6–10 days), resulting in increased ROS accumulation and decreased disease resistance. GC–MS analysis identified 87 metabolites (55 primary metabolites and 32 VOCs), revealing an increase in carbohydrates (glycerol, sorbitol) and a decrease in amino acids, organic acids, and heterocyclic compounds. These findings highlight a ROS-antioxidant-metabolite network underlying disease resistance, providing valuable insights for sustainable postharvest management.

## Highlights

• *N.dimidiatum* has significant impact on the terpenoids and esters of dragon fruit.
• Oxidative stress triggers ROS burst and antioxidant enzyme response.
• ROS-antioxidant-metabolite network forms dragon fruit defense system.
• Screened VOCs (geraniol, guaiacol) may help develop treatments for ulcer disease.

## 1 Introduction

Pitaya, also known as dragon fruit, is a tropical or subtropical fruit belongs to the cactus family, specifically the genera *Hylocereus* Britton & Rose or *Selenicereus* (A.Berger) Britton & Rose (Le Bellec & Vaillant, 2011). It is favored by consumers for its rich content of vitamins, dietary fiber, organic acids, amino acids, and sugars, as well as its sweet and delicate taste (Nishikito et al., 2023). However, the warm, humid climatic conditions conducive to pitaya growth also favors the germination, colonization, and proliferation of fungal pathogens (Intana, Kumla, Suwannarach, & Sunpapao, 2023). Dragon fruit canker disease, caused by the fungus *Neoscytalidium dimidiatum* (*N. dimidiatum*), poses a significant threat to dragon fruit plantations worldwide, leading to fruit rot, nutrient depletion, and off-flavors (Wang, Zhang, Yuan, & Chen, 2021). While chemical fungicides (e.g., pyraclostrobin) remain a primary control strategy, their long-term application risks pathogen resistance and chemical residue accumulation(Taguiam, Evallo, Bengoa, Maghirang, & Balendres, 2020). Consequently, there is a critical need for alternative approaches that leveraging the plant's innate defense mechanisms.

Understanding the physiological and biochemical changes in infected fruits is crucial for developing sustainable disease management strategies. These pathogen-induced alterations manifest as distinct metabolic signatures that can serve as diagnostic indicators. Such knowledge provides a theoretical foundation for early detection technologies by identifying characteristic metabolic markers before symptom manifestation (Prusky, Alkan, Mengiste, & Fluhr, 2013). Furthermore, elucidating fruit defense response mechanisms enables the development of green control technologies through induced resistance and modulation of metabolic pathways,offering alternatives to chemical fungicides (Romanazzi et al., 2016). Nevertheless, the metabolic responses of dragon fruit to *N. dimidiatum* infection and their potential for disease control remain largely unexplored.

When a plant is infected by a pathogen, it initiates a series of defense responses to inhibit pathogen growth, which include both physical and biochemical changes. Physical responses involve structural alterations such as cell wall thickening, callus formation, and the development of a cork layer (Chisholm, Coaker, Day, & Staskawicz, 2006). These changes are triggered by the regulation of primary metabolic pathways, including sugar metabolism, lipid metabolism, and amino acid metabolism. The physiological and biochemical responses of plants primarily involve the production of reactive oxygen species (ROS) and the synthesis of signaling compounds. Among these, the production of ROS, such as superoxide anion (O^2−^) and hydrogen peroxide (H_2_O_2_), represents a primary response of plant cells to pathogen invasion (Chen et al., 2020). Additionally, signaling molecules like salicylic acid (SA), jasmonic acid (JA), abscisic acid (ABA), and ethylene (ETH) play crucial roles in activating defense-responsive signaling pathways. These signaling molecules induce the expression of numerous defense genes, enabling the plant to mount a resistance response to pathogenic fungal stresses (Varela Alonso et al., 2022). Luna (2016) suggests that the plant pathogen defense mechanism functions similarly to a “green vaccine,” stimulating the plant's immune system to enhance its resistance. This mechanism has been demonstrated in “*Arabidopsis thaliana* (L.) Heynh”.

While ROS production represents the immediate cellular response, plants also activate secondary metabolite biosynthesis as a complementary defense strategy. Among plant defense secondary metabolites, volatile organic compounds (VOCs) act as chemical signals between plants and exhibit direct antimicrobial properties (Gershenzon, 2007). Recent research has demonstrated that plant VOCs, such as terpenes (isoprene, monoterpenes, and sesquiterpenes), phenylpropanoids, and fatty acid derivatives, have direct inhibitory effects on fungal pathogens and pests (Ricciardi et al., 2021). Studies on defense-related metabolites have provided valuable insights for developing sustainable disease management strategies. Research on *Artemisia annua* L. has demonstrated significant antioxidant, enzyme inhibitory and anti-inflammatory properties (Acquaviva et al., 2023), while investigations of *Arabis carduchorum* Boiss. have revealed substantial therapeutic potential through HPLC-MS/MS profiling (Uba et al., 2022). The documented antioxidant activity of *Coleus forskohlii* Briq. further supports the importance of plant secondary metabolites in defense responses (Takshak & Agrawal, 2015). For example, terpenoids exhibit strong anti-fungal activities (Sandargo, Michehl, Stadler, & Surup, 2019), while fatty acids effectively inhibit various plant pathogenic fungi (Walters, Raynor, Mitchell, Walker & Walker, 2004).

Despite these advances in understanding plant defense mechanisms, the specific pathogen-host interactions between dragon fruit and *N. dimidiatum*, particularly the metabolic responses and volatile profiles during postharvest infection, remain poorly understood. This study systematically analyzes the molecular mechanisms of pathogen-host interactions during the postharvest process of dragon fruit in response to *N. dimidiatum* infection, integrating gas chromatography-time-of-flight mass spectrometry (GC-TOF-MS) and headspace solid-phase microextraction-gas chromatography–mass spectrometry (HS-SPME-GC–MS) techniques. The research objectives are: (1) To characterize the spatiotemporal changes of primary metabolites (PM) and VOCs under pathogen infection stress, revealing the correlation between metabolic reprogramming and disease resistance phenotypes; (2) To explore the accumulation of ROS (O₂^−^, H₂O₂, MDA) and the response mechanisms of key antioxidant enzymes (SOD, CAT, POD, APX); (3) To construct an interaction network of ROS-antioxidant enzymes-defense metabolites (particularly VOCs with antimicrobial potential), elucidating the postharvest disease resistance response and its synergistic regulation mechanism. The research findings will provide new strategies for developing green postharvest disease control technologies based on targeted metabolite regulation, while also establishing a theoretical foundation for screening novel preservatives with dual functions of ROS scavenging and pathogen inhibition.

## 2 Materials and methods

### 2.1 Strain and dragon fruit preparation

This study isolated a strain of *N. dimidiatum* (GenBank accession no. PP946873) from a dragon fruit plantation in Luodian County, Qiannan Buyi and Miao Autonomous Prefecture, Guizhou Province. This strain is currently preserved at the College of Liquor and Food Engineering, Guizhou University. The strain was inoculated onto potato dextrose agar and incubated at 28 °C for 7 d. During this period, the mycelium on the surface of the medium gradually turned black.

Freshly ripened dragon fruits (30–40 d after pollination) were collected from Luodian County, Qiannan Buyi and Miao Autonomous Prefecture, Guizhou Province, and transported to the laboratory at a low temperature within 2 h. Only dragon fruits that were free of rot and damage, and uniform in size, color, and shape, were selected for the subsequent experiments.

### 2.2 Pathogenicity tests for pathogenic Bacteria

The surface of the fruit was sprayed with a 0.5 % sodium hypochlorite solution for 10 s, rinsed with sterile water, and then air-dried. This process was repeated three times to eliminate surface microorganisms. Small holes (0.5 mm in diameter and 2 mm deep) were punched into 120 sterilized dragon fruits, which were then divided into two groups: a control group (ck) and a group inoculated with *N. dimidiatum* mycelium (rno) (For the rno group, actively growing mycelium scraped from 7-day-old PDA cultures was packed into each wound (0.3–0.5 mg wet weight per wound). The ck group wounds were left untreated). The fruits were stored in sterile boxes at a temperature of (28 ± 2 °C and 85 ± 5 % relative humidity), with physicochemical tests conducted at 0, 2, 4, 6, 8, and 10 days. Pulp samples were collected at 0, 6, and 10 days, pooled for biological replicates, frozen in liquid nitrogen, and stored at −80 °C for metabolite analysis.

### 2.3 Measurement of fruit quality indices

#### 2.3.1 Respiration rate

The respiration rate of dragon fruit was measured using a respirometer (JC-FS-3080 A, Qingdao Juchuang). A single fruit was sealed in a plastic airtight container at 25 °C, and CO2 concentration changes were detected. Each fruit was tested three times, with a total of three fruits. The respiration rate was expressed as mg CO_2_ kg^−1^ h^−1^ FW (fresh weight).

#### 2.3.2 Total soluble solids (TSS) and titratable acidity (TA)

The TSS of dragon fruit were measured using a refractometer (LTY330) and expressed as °Brix (%). The TA was determined by titration with 0.1 M NaOH to a pH of 8.2, and expressed as citric acid percentage. Each measurement was repeated three times.

#### 2.3.3 Weight loss rate

To measure weight loss during storage, the weights of dragon fruits on 2, 4, 6, 8, and 10 d were compared to the initial weight recorded at 0 d. The weight loss rate of the fruit was calculated using formula (1):

(1) $$\text{Weight loss rate}=\frac{\text{Initial weight}-\text{weighing weight}}{\text{initial weight}}\times 100\%$$

#### 2.3.4 Hardness

The hardness of dragon fruit at different storage stages was measured using a texture analyzer (TA.TOUCH, Shanghai Baosheng). Fruit slices (20 mm thick) were compressed with a 2 cm aluminum probe. The test parameters: pre-test speed 1 mm s^−1^, test speed 0.3 mm s^−1^, post-test speed 1 mm s^−1^, 2 s interval. Three fruits were tested at each stage, with three measurements per fruit, and the average compression force (N) was recorded.

### 2.4 ROS and MDA measurement

#### 2.4.1 O₂^−^ generation rate measurement

Three grams of frozen dragon fruit peel powder were homogenized with 15 mL of 50 mM sodium phosphate buffer (pH 7.8) containing 1 mM EDTA, 1 % PVP (*w*/*v*), and 0.3 % Triton X-100. The mixture was centrifuged at 5000×*g* for 15 min at 4 °C. Then, 0.5 mL of the supernatant was mixed with 1.0 mL of 100 mM sodium phosphate buffer (pH 7.8) and 0.5 mL of 10 mM hydroxylamine hydrochloride, and reacted at 25 °C for 20 min. Next, 1 mL of 17 mM sulfanilic acid and 1 mL of 7 mM α-naphthylamine were added, and the reaction continued for another 20 min at 25 °C. After adding 4 mL of n-butanol for extraction, the absorbance of the n-butanol phase was measured at 530 nm. The O₂^−^ generation rate was expressed as μmol kg^−1^ min^−1^.

#### 2.4.2 H₂O₂ content measurement

Three grams of frozen tissue were homogenized in 3 mL of cold acetone and centrifuged at 9000×*g* for 20 min at 4 °C. One milliliter of the supernatant was mixed with 200 μL of titanium tetrachloride and allowed to settle at 4 °C. After centrifugation at 9000×*g* for 10 min, the precipitate was dissolved in 3 mL of 1 M H₂SO₄, and the absorbance was measured at 410 nm. The H_2_O_2_ content was expressed as μmol kg^−1^ FW.

#### 2.4.3 MDA content measurement

Three grams of fruit peel powder were mixed with 15 mL of 5 % trichloroacetic acid (TCA) and centrifuged at 8000×*g* for 10 min at 4 °C. One milliliter of the supernatant was mixed with 3 mL of 0.5 % thiobarbituric acid (TBA) in 10 % TCA and reacted in a boiling water bath for 20 min, followed by ice bath cooling. After centrifugation at 8000×*g* for 10 min, the absorbance of the supernatant was measured at 532 nm (background at 600 nm subtracted). MDA content was calculated using an extinction coefficient of 155 mM^−1^ cm^−1^ and expressed as nmol g^−1^ FW.

### 2.5 Antioxidant enzyme activity measurement

Three grams of liquid nitrogen-frozen and ground fruit peel tissue were homogenized with the following precooled (4 °C) buffer solutions to prepare enzyme extracts:

POD (EC 1.11.1.7): 15 mL of 50 mM sodium phosphate buffer (pH 7.0) containing 5 % (*w*/*v*) PVP.
SOD (EC 1.15.1.1) and CAT (EC 1.11.1.6): 15 mL of 100 mM sodium phosphate buffer (pH 7.5) containing 5 % (w/v) PVP and 5 mM dithiothreitol.
APX (EC 1.11.1.11): 15 mL of 100 mM potassium phosphate buffer (pH 7.5) containing 2 % PVP, 1 mM ascorbic acid, and 1 mM EDTA.

The homogenate was centrifuged at 12,000×*g* for 20 min at 4 °C, and the supernatant was collected for enzyme activity analysis.

#### 2.5.1 POD activity measurement

Forty microliters of enzyme extract were mixed with 10 μL of 0.5 M H₂O₂ and 150 μL of 25 mM guaiacol solution. The absorbance was continuously measured at 470 nm for six intervals (1 min each). The enzyme activity unit (U) is defined as the amount of enzyme that causes a 0.01 increase in absorbance per minute, with the calculation formula: U = 0.01ΔOD₄₇₀/min.

#### 2.5.2 SOD activity measurement

The reaction system contained 100 μM EDTA, 130 mM methionine, 50 mM sodium phosphate buffer (pH 7.8), 750 μM nitro blue tetrazolium (NBT), 20 μM riboflavin, and crude enzyme solution. After reaction in the dark, the absorbance was measured at 560 nm. The enzyme activity is expressed as 1 unit (U) per 50 % inhibition of NBT reduction, with the calculation formula: U = 0.001ΔOD₅₆₀/min.

#### 2.5.3 CAT activity measurement

Three hundred microliters of crude enzyme solution were mixed with 3 mL of 0.01 M H_2_O_2_. The absorbance decrease at 240 nm was immediately measured. The enzyme activity was calculated using the formula: U = 0.01ΔOD₂₄₀/min.

#### 2.5.4 APX activity measurement

The reaction system contained 0.2 mL of enzyme solution, 2 mL of 100 mM sodium phosphate buffer (pH 7.5), and 0.5 mL of 30 % H_2_O_2_. The change in absorbance at 340 nm was measured, and the enzyme activity was calculated using the formula: U = 0.01ΔOD₃₄₀/min.

### 2.6 Primary metabolite composition

PMs were analyzed using GC-TOF-MS, Approximately 25 mg of the sample was placed in a 2 mL tube containing 1000 μL of extraction solution (methanol:water = 3:1, with ribitol as the internal standard) and steel beads. The sample was ground, sonicated, frozen, and then centrifuged. The supernatant was concentrated, dried, and derivatized using methoxamine hydrochloride and BSTFA. Analysis was performed using GC-TOF-MS with a DB-5MS column, operating in splitless mode and utilizing helium as the carrier gas. The oven temperature was ramped from 50 °C to 310 °C, and the mass range was set to m z^−1^ 50–500.

### 2.7 Volatile compounds

#### 2.7.1 Detection of VOCs in dragon fruit pulp

The determination of VOCs was performed following and modifying the method established by (Gong et al., 2023). Specifically, Samples were ground in liquid nitrogen, and 500 mg of each sample was placed in a 20 mL vial containing a NaCl solution and an internal standard. After sealing, the vial was shaken at 60 °C for 5 min. A 120 μm SPME Arrow was used for 15 min of solid-phase extraction, followed by GC–MS/MS analysis at 250 °C for 5 min. The oven temperature program was: 40 °C for 3.5 min, ramped to 100 °C, then 180 °C, and finally 280 °C. VOCs were identified using selected ion monitoring (SIM) mode with helium as the carrier gas.

#### 2.7.2 Analysis of relative odor activity value (rOAV_*i*_) of VOCs

The relative odor activity value (rOAVi) assesses the contribution of key flavor compounds to a food's aroma based on their sensory threshold. An rOAVi ≥1 indicates a direct contribution to the flavor. The calculation is shown in formula (2):

(2) $$\mathrm{rOAVi}=\frac{\mathrm{Ci}}{\mathrm{Ti}}$$

where rOAV_*i*_ represents the relative odor activity value of compound *i*, *Ci* represents the relative content of the compound (μg g^−1^ or μg mL^−1^), and *Ti* represents the threshold of the compound (μg g^−1^ or μg mL^−1^).

### 2.8 Amino acids

Approximately 1 g of dragon fruit pulp was extracted using 50 mL of 0.01 N hydrochloric acid for 30 min, then filtered. A 2 mL of the filtrate was mixed with 2 mL of 8 % sulfosalicylic acid, allowed to stand for 15 min, and then centrifuged at 10,000 rpm for 10 min. The supernatant was filtered through a 0.22 μm membrane and transferred to a sample vial. The free amino acid content was measured using an amino acid analyzer (Sykam S-433D, Germany).

### 2.9 Metabolite identification and data analysis

Mass spectrometry data were processed using ChromaTOF (V4.3×, LECO) for peak extraction, baseline correction, deconvolution, integration, and alignment. PMs were identified with the LECO-Fiehn Rtx5 database. Peaks with detection rates <50 % or RSDs >30 % in QC samples were excluded. Data preparation included missing value imputation (half minimum method) and TIC normalization.

The significance of differences was determined using a one-way analysis of variance (ANOVA) conducted with SPSS v25.0 (IBM, Armonk, NY, USA), and Tukey's test (*P* < 0.05) was used to identify significant differences. Metabolite data were subjected to partial least squares discriminant analysis (PLS-DA) through the metabolomics analysis tool (https://www.metaboanalyst.ca/). KEGG Enrichment Analysis: KEGG pathway annotation of the differential abundance metabolites was performed based on their cpd_IDs, and the *p*-value for each pathway was calculated using hypergeometric testing. The Rich factor represents the ratio of the number of differentially expressed metabolites in each pathway to the total number of metabolites detected and annotated in that pathway.

## 3 Results

### 3.1 Effects of *N. dimidiatum* on fruit quality of dragon fruit

**Fig. 1:**
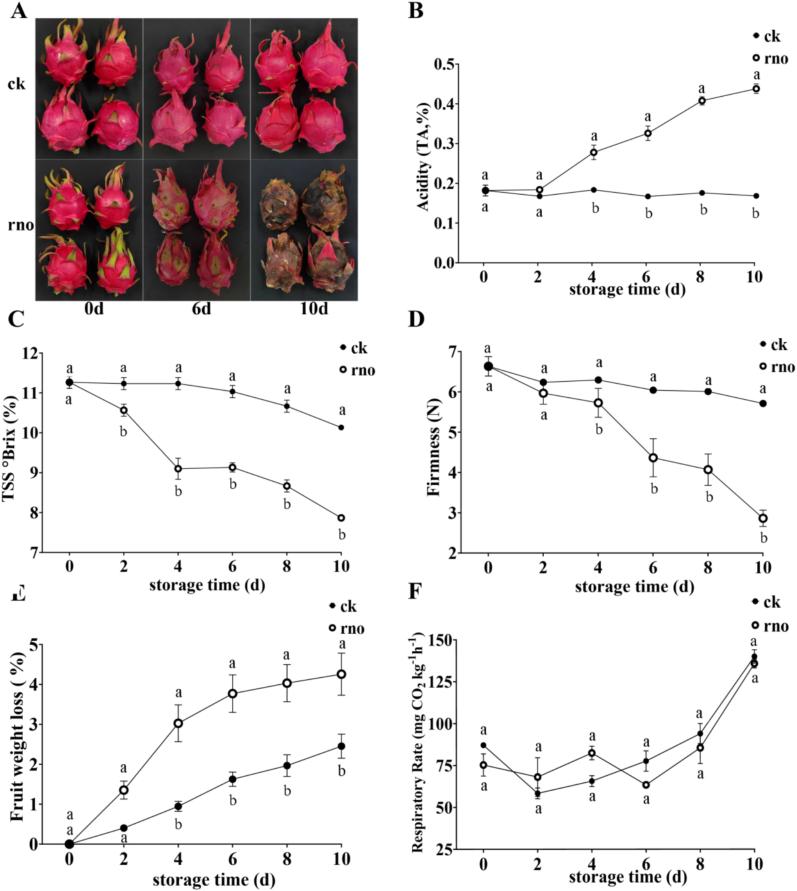
The effects of infection with *N. dimidiatum* on the appearance (A), TA content (B), TSS content (C), firmness (D), fruit weight loss rate (E), and respiration rate (F) of pitaya were studied. Data are presented as mean ± standard error (*n* = 3). different letters show significant difference according to least significant difference test at *P <* 0.05 .

The dragon fruits in the ck group remained disease-free throughout storage (10 d), while the rno group developed large yellow-brown spots starting on day 6 and were fully decayed by day 10, emitting a foul odor (Fig. 1A). The TA content in the rno group increased significantly, from 0.18 % ± 0.01 % on day 0 to 0.42 % ± 0.01 % on day 10, while the ck group remained stable (*P* < 0.05) (Fig. 1B). The TSS content in the rno group decreased from 11.27°Brix (%) ± 0.15°Brix (%) to 7.87°Brix (%) ± 0.06°Brix (%), which is 2.27°Brix (%) lower than that of the ck group (Fig. 1C).

The flesh hardness of dragon fruit in both the ck and rno groups decreased during storage. However, the rno group, inoculated with *N. dimidiatum*, showed a more rapid decline starting from day 4. By day 10, the hardness of the rno group had decreased to 2.86 N, which was 2.85 % lower than that of the ck group (*P* < 0.05) (Fig. 1D). Similarly, the weight loss rate was higher in the rno group, with a maximum loss of 4.26 ± 0.53 % on day 10, compared to 2.45 ± 0.30 % in the ck group (*P* < 0.05) (Fig. 1E). The respiration rates in both groups increased from day 6, peaking at day 10 (rno = 135.77 ± 2.62 mg CO_2_ kg^−1^ h^−1^, ck = 140 ± 4.01 mg CO_2_ kg^−1^ h^−1^) (Fig. 1F).

### 3.2 Impact of *N. dimidiatum* inoculation on ROS and MDA levels during dragon fruit storage

**Fig. 2:**
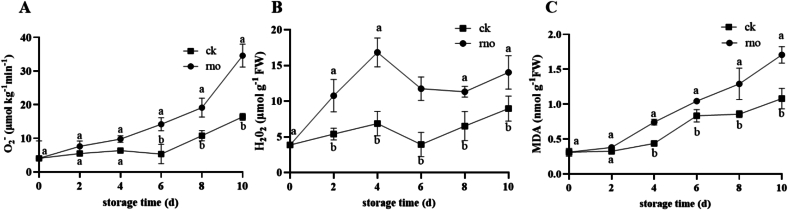
Changes in O₂^−^ (A), H₂O₂ (B), and MDA (C) contents in *N. dimidiatum*-infected (rno) and healthy pitaya (ck) during storage (0–10 days). Data are presented as mean ± standard error (n = 3). Different letters (a, b) indicate significant differences between rno and ck groups at the same time point (*P* < 0.05).

Compared to the control group (ck), dragon fruit infected with *N. dimidiatum* (rno group) exhibited a significant increase in reactive oxygen species (ROS) and lipid peroxidation levels during storage. Specifically, the O₂^−^ and MDA contents in the rno group continuously increased over the storage period, reaching 2.11 and 1.58 times those of the ck group by day 10 (Fig. 2A, C), while the ck group maintained low, fluctuating levels. The H₂O₂ content in the rno group increased in a fluctuating manner but remained significantly higher than in the ck group (Fig. 2B). These results indicate that *N. dimidiatum* inoculation accelerates oxidative damage in dragon fruit.

### 3.3 Impact of *N. dimidiatum* inoculation on antioxidant enzyme (SOD, CAT, POD, APX) activity during dragon fruit storage

**Fig. 3:**
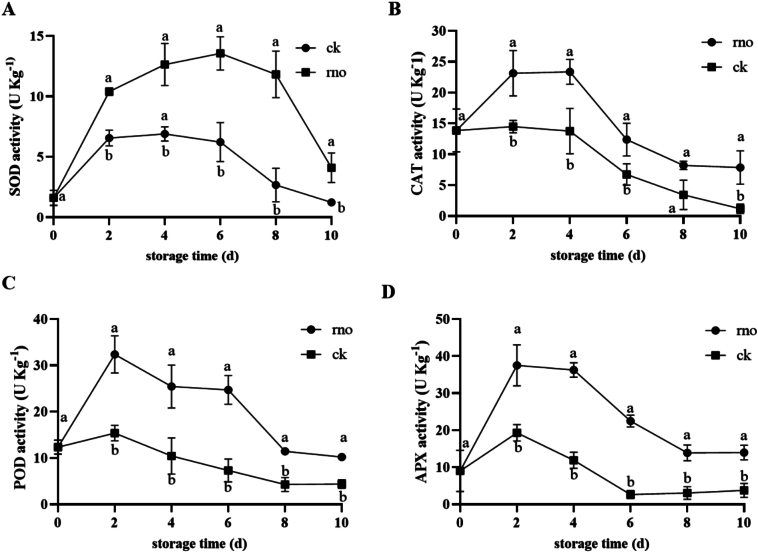
Changes in the activities of superoxide dismutase (SOD) (A), catalase (CAT) (B), peroxidase (POD) (C), and ascorbate peroxidase (APX) (D) in *N. dimidiatum-*infected (rno) and healthy pitaya (ck) during storage (0–10 days). Data are presented as mean ± standard error (n = 3). Different letters (a, b) indicate significant differences between the rno and ck groups at the same time point (*p* < 0.05).

As shown in Fig. 3, *N. dimidiatum* inoculation significantly regulated changes in dragon fruit antioxidant enzyme activity. During the early storage period (0–2 days), the activity of SOD, CAT, POD, and APX in the rno group was significantly higher than in the ck group (*P* < 0.05). Specifically, SOD activity peaked on day 6 (13.56 ± 1.22 U·kg^−1^, 2.18 times that of the ck group, Fig. 3A), while CAT activity remained high from day 2 to day 4 (23.13 ± 3.07 to 23.37 ± 1.74 U·kg^−1^, Fig. 3B). POD and APX activity were 2.1 times and 1.94 times that of the ck group on day 2, respectively (Fig. 3C, D). In the later storage period (6–10 days), all enzyme activities in the rno group continuously decreased, reaching their lowest levels by day 10, while the ck group maintained low, fluctuating activity levels. These results suggest that pathogen inoculation activates the antioxidant enzyme system early in the storage period, but as the disease progresses, enzyme activity is significantly suppressed due to metabolic disorder or depletion of defense resources.

### 3.4 Primary metabolic profiling

**Fig. 4:**
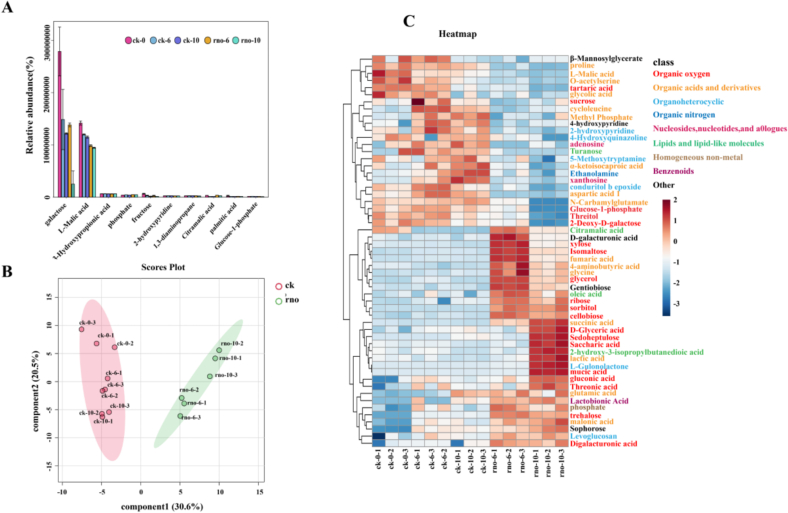
Changes in the relative content of the top 10 PMs in dragon fruit (A); Principal component analysis (PCA) of primary metabolite changes in the flesh of dragon fruit between the two groups (B); Heatmap of differential PMs (VIP > 1, *P* < 0.05; colors used to differentiate categories) (C).

This study identified 141 PMs in the flesh of dragon fruit from both groups (Table S1). Galactose and L-malic acid were the most prevalent, comprising 46.95 % and 37.55 % of the total PMs, respectively (Fig. 4A). Both metabolites decreased over the storage period in both the ck and rno groups, with a more significant reduction observed in the rno group.

To assess the impact of *N. dimidiatum* on PMs during dragon fruit storage, we performed principal component analysis (PCA) on the PMs from both dragon fruit groups (ck and rno) (Fig. 4B). Components 1 (30.6 %) and 2 (20.5 %) explained 51.1 % of the variance, with significant separation between the rno and ck groups, suggesting pathogen invasion affected primary metabolite production or consumption. A total of 55 differential PMs were identified (VIP > 1, *P* < 0.05).

#### 3.4.1 Changes in differential organic oxidative compounds in dragon fruit flesh post-canker disease

We identified 20 differential organic oxidative compounds from the metabolites of the two dragon fruit groups (ck and rno) (Fig. 4C), all of which belonging to carbohydrates and carbohydrate conjugates. In both groups, the relative contents of 5 carbohydrate compounds decreased over time, with a more significant reduction in the rno group. For example, after 10 days, the levels of tartaric acid, threitol, glucose-1-phosphate, and 2-deoxy-D-galactose in the ck group were about 1.5 times higher than those in the rno group. Additionally, 15 carbohydrate compounds in the rno group showed a significant increase in relative content during storage, predominantly sugar alcohols and sugar acids.

#### 3.4.2 Changes in differential organic acids and their derivatives in dragon fruit flesh post-canker disease

In Fig. 4C, 16 differential organic acids and their derivatives were identified. Among these, glycine, fumaric acid, 4-aminobutyric acid, malonic acid, succinic acid, lactic acid, and glutamic acid exhibited abnormal accumulation in the rno group. Specifically, glycine, 4-aminobutyric acid, and fumaric acid reached their peak relative contents at 6 d of storage in the rno group, while the others peaked at 10 d of storage. The remaining 9 differential organic acids showed varying degrees of reduction in relative content in the rno group compared to the ck group.

#### 3.4.3 Changes in differential organic heterocyclic compounds, lipids, and lipid-like molecules in dragon fruit flesh post-canker disease

In addition to the differential PMs, 3 lipids, 2 organoheterocyclic compounds, 1 non-metal compound, 1 benzenoid, and 3 other metabolites showed varying accumulation in the rno group (Fig. 4C). Gentiobiose, citramalic acid, phosphate, oleic acid, and D-galacturonic acid peaked at 6 d, while sophorose, 2-hydroxy-3-isopropylbutanedioic acid, and gulonolactone peaked at 10 d. Conversely, 4 organoheterocyclic compounds, 1 lipid-like molecule, 2 nucleosides, 1 organic nitrogen compound, and 2 other compounds decreased, including 4-hydroxyquinazoline, 5-methoxytryptamine, adenosine, xanthosine, and ethanolamine.

#### 3.4.4 KEGG pathway analysis of differential metabolites

**Fig. 5:**
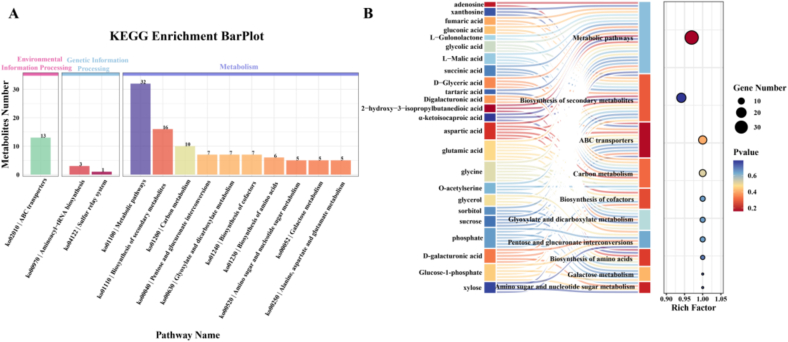
KEGG Pathway Enrichment Analysis of Differential Metabolites (top10) (A). Bubble plot illustrating the KEGG pathway enrichment of differential metabolites, highlighting the top 10 pathways (B).

A KEGG enrichment analysis was performed to explore the biological pathways enriched with differential PMs during the storage of dragon fruit. As shown in Fig. 5A, most metabolites were enriched in the “Metabolism” category, while fewer were found in “Environmental Information Processing” and “Genetic Information Processing.” The analysis identified 66 metabolic pathways associated with these differential metabolites (Fig. 5B). Key pathways involved include “Metabolic Pathways,” “Biosynthesis of Secondary Metabolites,” and “Carbon Metabolism,” all of which play crucial roles in the synthesis of these metabolites and are highly active during plant-pathogen interactions.

### 3.5 Volatile compounds

**Fig. 6:**
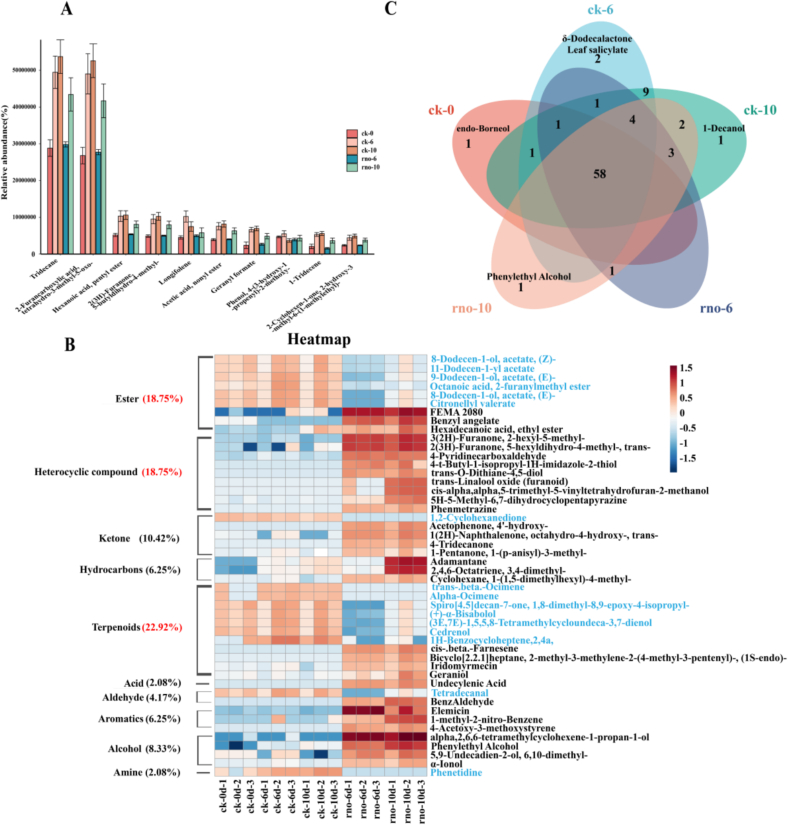
Relative content of the top 10 VOCs in dragon fruit (A); Heatmap of differential compounds (VIP > 1, *P* < 0.05, |log₂(FC)| > 2; substances with decreased relative content are indicated in blue text) (B); Venn diagram of rOAV values for dragon fruit (C). (For interpretation of the references to color in this figure legend, the reader is referred to the web version of this article.)

In this study, 785 metabolites were identified in dragon fruit pulp using HS-SPME-GC–MS, including 196 terpenes, 148 esters, 109 heterocyclic compounds, 58 ketones, 52 alcohols, and other compounds (Table S2). As illustrated in Fig. 6A, tridecane and 2-furancarboxylic acid, tetrahydro-3-methyl-5-oxo- were the most abundant VOCs, accounting for 14.57 % and 14.04 %, respectively, significantly surpassing hexanoic acid, pentyl ester (2.81 %). In the rno group, the relative content of the top 10 VOCs consistently increased during storage. In contrast, in the ck group, longifolene and phenol, 4-(3-hydroxy-1-propenyl)-2-methoxy- peaked on day 6, with the other top 10 compounds gradually increased over time.

A total of 32 differential VOCs were identified between the two groups, including terpenes (22.92 %), esters (18.75 %), organohalogen compounds (18.75 %), and others (Fig. 6C). The rno group exhibited 16 compounds with lower relative content, mainly esters and terpenes, while 16 compounds, mainly organohalogen compounds and ketones, showed increased relative content compared to the ck group.

#### 3.5.1 Changes in differential terpenes and esters in dragon fruit flesh

Longifolene and hexanoic acid, pentyl ester, are the most abundant VOCs in both groups of dragon fruit, accounting for 2.37 % and 2.81 % of the total VOCs, respectively (Fig. 6A). Terpenoids represent the most prevalent differential VOCs, with 11 identified compounds (Fig. 6B), followed by esters and organohalogens, each containing 9 compounds. Infected dragon fruit showed increased levels of four terpenes: bicyclo[2.2.1]heptane, cis-β-farnesene, iridomyrmecin, and geraniol. Conversely, seven terpenes, including trans-β-ocimene and α-ocimene, showed a decrease in relative content post-infection. Notably, five terpenes showed an initial decrease, followed by an increase, with the lowest levels on day 6. 6 differential ester compounds also exhibited a similar trend, with compounds like 8-dodecen-1-ol acetate and citronellyl valerate initially decreasing and then increasing. In contrast, FEMA 2080, benzyl angelate, and hexanoic acid, pentyl ester increased, particularly FEMA 2080 and benzyl angelate (log2(FC) = 5.75 and 4.62, respectively).

#### 3.5.2 Changes in differential heterocyclic compounds and ketones in dragon fruit flesh

Among the differential VOCs in the ck and rno groups, 9 heterocyclic compounds (18.75 %) and 5 ketones (10.42 %) were identified. The relative content of all differential heterocyclic compounds increased after infection. Notably, 3-(2H)-furanone, 2-hexyl-5-methyl- (log2(FC) = 6.09) and 2-(3H)-furanone, 5-hexyldihydro-4-methyl-, trans- (log2(FC) = 5.21) showed significant increases. In contrast, among the ketones, 1,2-cyclohexanedione showed a decrease in relative content, while the other four ketones exhibited increases.

#### 3.5.3 Changes of differential alcohols and hydrocarbons in dragon fruit pulp

Following canker disease infection, the relative content of 4 alcohols, 3 aromatics, 3 hydrocarbons, 1 aldehyde, and 1 acid increased. Significant increases were observed in alpha,2,6,6-tetramethylcyclohexene-1-propan-1-ol (log2(FC) = 8.10), phenylethyl alcohol (log2(FC) = 4.98), elemicin (log2(FC) = 7.26), and benzaldehyde (log2(FC) = 4.08). Conversely, phenetidine decreased, following a similar trend as terpenes and esters.

#### 3.5.4 rOAV_*i*_ analysis of aroma active compounds

In this study, rOAV*i* values were calculated for 85 VOCs, with values ≥1 indicating a contribution to aroma. Unique compounds include *endo*-borneol in the ck-0 group, 1-decanol in the ck-10 group, phenylethyl alcohol in the rno-10 group, and δ-dodecalactone and cis-3-hexenyl salicylate in the ck-6 group. 58 VOCs are shared between both groups. Key aroma contributors with rOAV*i* values over 700 include benzenemethanethiol, 2(5H)-furanone, 6-nonenal (*Z*)-, and phenylethyl isovalerate.

### 3.6 Free amino acid analysis

**Fig. 7:**
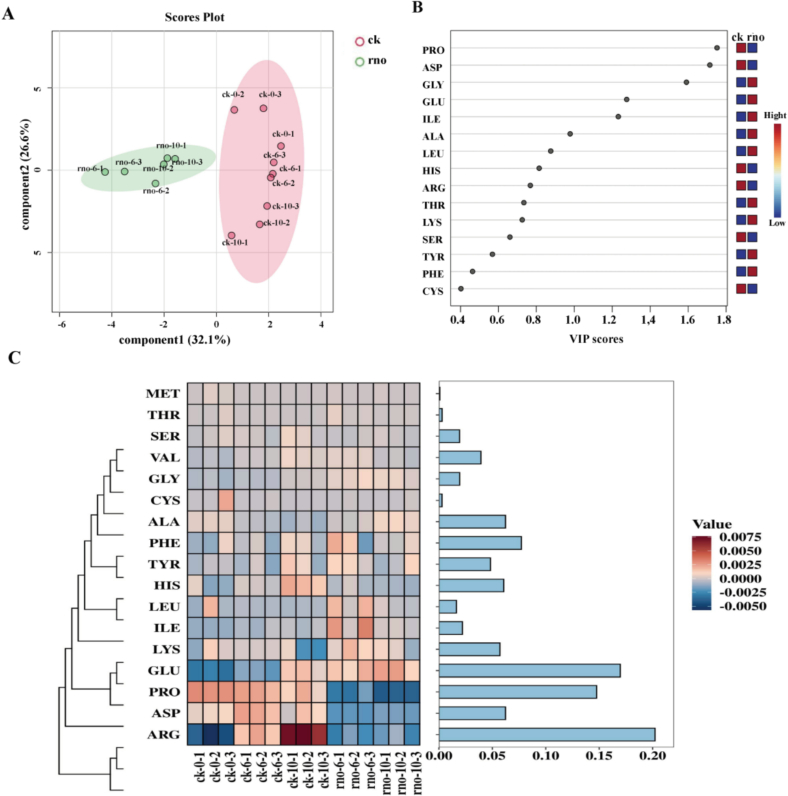
PCA Analysis of Relative Content of Free Amino Acids (A); VIP Scores (B); Cluster Heatmap of Free Amino Acids (C).

The study quantified 17 free amino acids in dragon fruit flesh during storage, using an internal standard method. Principal Component Analysis (Fig. 7A) showed significant separation between the rno and ck groups, accounting for 58.7 % of the total variance, which indicates notable differences in amino acid composition. The most abundant amino acids in both groups were Arginine (ARG) (20.02 %), Glutamic Acid (GLU) (16.79 %), and Proline (PRO) (14.61 %) (Fig. 7C). During storage, ARG increased in the ck group, while PRO decreased in the rno group. GLU accumulated mainly in the ck-10 and rno groups. Differential amino acids (VIP > 1) included PRO, ASP, GLY, GLU, and ILE (Fig. 7B). After pathogen inoculation, PRO and ASP decreased, while GLY, GLU, and ILE increased.

## 4 Discussion

### 4.1 Impact of pathogens on dragon fruit quality

Our study found that dragon fruit deteriorates during natural storage after harvest, resulting in rapid declines in freshness and quality. This deterioration includes softening, off-flavors, increased weight loss and respiration rates, as well as changes in physicochemical indicators, such as decreases in TSS and TA, consistent with previous research (Li, Deng, Shen, Zhang, & Sun, 2024). However, the presence of plant pathogens significantly shortens the storage and shelf life of dragon fruit (Pan, Zhang, Lu, Liu, & Fu, 2021). In this study, fresh dragon fruit infected with canker disease showed exacerbated deterioration in all indicators, except for respiration rate, compared to the ck group. Notably, significant changes in post-harvest physiological indicators often result from metabolic responses to environmental changes during storage. Compared to the control group, the TA content in the rno group significantly increased, while the TSS content drastically decreased, imparting an overall acidic taste to the dragon fruit. This distinct acidification pattern differs from the neutral pH maintenance observed in citrus-*Penicillium* interactions (Prusky, McEvoy, Saftner, Conway, & Jones, 2008). This change may be attributed to the excessive accumulation of key organic acids, such as malic acid, citric acid, and fumaric acid in the red-fleshed dragon fruit of the rno group (Angonese et al., 2021). Furthermore, our study found that pathogens not only affect the flavor and taste of dragon fruit but also accelerate its softening and weight loss. Pathogens produce a range of plant cell wall-degrading enzymes, including cellulases, hemicellulases, and pectinases (Glass, Schmoll, Cate, & Coradetti, 2013). These enzymes break down plant tissues, and the resulting monomers and/or small oligosaccharides are transported into the cells via membrane-bound transport proteins to support fungal growth and reproduction. This process ultimately leads to cell rupture, juice leakage, and moisture loss, thereby exacerbating fruit softening and deformation.

### 4.2 Defensive response of dragon fruit PMs to pathogens

Plant-synthesized PMs, such as carbohydrates, proteins, and lipids, not only support normal growth and development but also play a crucial role in plant responses to biotic stress. Upon infection, the metabolic pathways in dragon fruit are altered, leading to a redistribution of carbon, nitrogen, and energy among various metabolic pathways. The regulation of carbon metabolism pathways increases the synthesis of soluble sugars like cellobiose, trehalose, and isomaltose, providing dragon fruit with extra energy and carbon sources. This supports defense responses, tissue repair, and the production of antimicrobial substances such as trehalose (Formela-Luboińska et al., 2020). Furthermore, sugar alcohols (such as glycerol and sorbitol) can rapidly provide energy by being converted into other carbohydrates or entering glycolysis. They also act as antioxidants to reduce oxidative stress and protect plant cells from damage caused by fungal infections (Gine-Bordonaba et al., 2020). Dragon fruit reduces the synthesis of storage sugars, such as sucrose, redirecting resources toward defense responses. This shift contributes to the decrease in TSS content after infection. The study found that amino acid biosynthesis pathways are crucial for the fruit's physiological and defensive mechanisms. Following infection, dragon fruit regulates these pathways, using proline (PRO) for signal transduction and as an antioxidant to scavenge ROS generated by the pathogen (Thangaraj et al., 2022), ASP may be consumed as a precursor for the synthesis of plant hormones (such as ethylene) and for strengthening cell walls (Han et al., 2024). GLY and GLU have long been recognized as key components of plant immune responses. In dragon fruit, their accumulation following infection activates the expression of a range of immune-related genes, including those encoding disease-resistant proteins, antioxidant enzymes, and signaling molecules (Jiang et al., 2017). ILE also accumulates in dragon fruit following infection. Its role in plant defense is still under investigation, but it may be involved in synthesizing secondary metabolites and regulating growth and resistance.

Secondary metabolite biosynthesis pathways play a vital role in activating plant defenses. Following an infection, dragon fruit rapidly converts stored precursors into defensive metabolites, including isoprenoids, phenylpropanoids, and organic acids. This process is catalyzed by enzymes such as CoA, IPP, and TrpS. Compounds like cis-β-farnesene, geraniol, α,2,6,6-tetramethylcyclohexene-1-propan-1-ol, and fumaric acid demonstrate antifungal properties, enhancing the fruit's self-defense mechanisms(Xie et al., 2023). In addition, the biosynthesis of auxiliary factors, the metabolism of acetylsalicylates and dicarboxylates, the interconversion of pentoses and glucuronic acid, and the metabolism of alanine, aspartic acid, and glutamic acid, as well as the regulation of amino sugar and nucleotide sugar metabolism, are also associated with the activation of plant defenses. These processes collectively contribute to providing energy and carbon sources during the defense response in dragon fruit, synthesizing defensive compounds such as phenolic substances and antioxidants, and producing structural molecules (Pinheiro et al., 2022).

### 4.3 Volatile metabolites and ROS-antioxidant enzyme synergistic defense network in dragon fruit

**Fig. 8:**
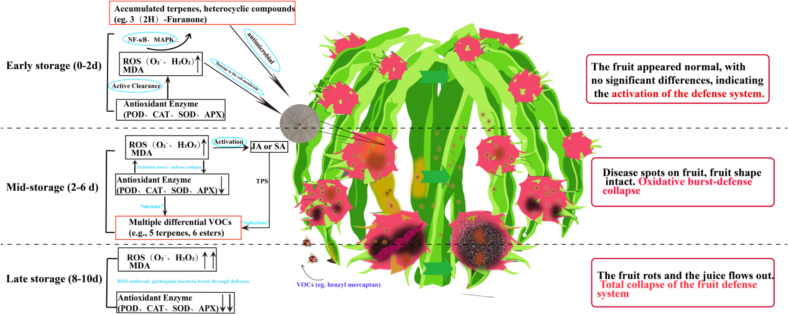
Dynamic changes in volatile metabolites (VOCs) and the synergistic defense mechanism of the ROS-antioxidant enzyme system in response to *N. dimidiatum* infection in dragon fruit.

This study reveals the dynamic changes in volatile organic compounds (VOCs) and the synergistic defense characteristics of the ROS-antioxidant enzyme system in response to *N. dimidiatum* infection in dragon fruit (Fig. 8). First, pathogen inoculation significantly induces a rapid accumulation of ROS (O₂^−^, H₂O₂), along with a continuous increase in MDA levels(Fig. 2A–C), indicating that oxidative stress is a core driving force in the progression of dragon fruit canker disease. Notably, excessive ROS accumulation has a dual effect: on one hand, it directly damages the cell membrane system (indicated by increased MDA); on the other hand, it may act as a signaling molecule to activate secondary metabolic pathways (Fig. 3B)(Mittler et al., 2011). This dual-role mechanism advances the current understanding of ROS function beyond the traditional “oxidative damage” paradigm established in plant stress biology (Sood, 2025), demonstrating that ROS act as both destructive and constructive forces in postharvest defense. This could be linked to the significant changes in VOCs (such as terpenes and heterocyclic compounds) in the rno group (Fig. 3B). For example, 3(2H)-Furanone and 2(3H)-Furanone show log2(FC) values of 6.09 and 5.21, respectively (Section 3.5.2), and their biosynthesis might be regulated by H₂O₂-mediated redox signaling(Farmer & Mueller, 2013), with these compounds having confirmed antimicrobial activity(PAULITZ, NOWAK-THOMPSON, GAMARD, TSANG & LOPER Paulitz, Nowak-Thompson, Gamard, Tsang, & Loper, 2000).

Secondly, the dynamic response of the antioxidant enzyme system further underscores the key role of redox balance in plant defense. During the early storage period (0–2 days), the activities of SOD, CAT, POD, and APX in the rno group were significantly higher than those in the ck group (Fig. 3D), suggesting that pathogen infection triggers the host's active ROS scavenging mechanism to delay oxidative damage. However, by the later storage period (6–10 days), enzyme activity sharply declined (with SOD activity dropping by 62.5 %), suggesting that ROS accumulation exceeded the antioxidant system's regulatory threshold, leading to a vicious cycle of “oxidative burst-defense collapse-metabolic rebound.” This turning point (day 6) coincides with the “decrease-then-increase” trend in the relative content of multiple differential VOCs (such as 5 terpenes and 6 esters) (Section 3.5.1). The possible mechanism is that early antioxidant enzymes temporarily suppress secondary metabolite synthesis by scavenging ROS (Myers, Fichman, Zandalinas, & Mittler, 2023), while the subsequent enzyme activity failure led to ROS re-accumulation, activating jasmonic acid (JA) or salicylic acid (SA) signaling pathways (Santino et al., 2013), which in turn drove the expression of terpene synthase (TPS) and esterase genes, resulting in the rebound accumulation of related VOCs (Yang et al., 2025).

Plant volatile metabolites, through their synergistic action with the antioxidant system, construct a multi-layered defense network: on one hand, upregulated terpenes (such as geraniol), furans, and phenolic compounds (such as eugenol) exert direct inhibitory effects by disrupting pathogen cell membrane integrity or inhibiting spore germination; on the other hand, volatile organics like benzaldehyde and phenylethanol not only have the ability to scavenge free radicals to alleviate oxidative stress(Mittler, 2002), but their high-aroma active compounds (e.g., benzyl mercaptan, isoamyl acetate) can trigger systemic resistance in neighboring tissues via gaseous signaling molecules(Kessler, Mueller, Kalske, & Chautá, 2023), forming a “direct inhibition - oxidative regulation - systemic defense” synergistic defense system. This multi-functional VOCs concept advances beyond traditional single-target antimicrobial research toward a comprehensive understanding of integrated defense molecule.

In conclusion, dragon fruit constructs a dynamic defense network through the “oxidative burst-defense collapse-metabolic rebound” cascade (Fig. 8). However, pathogens ultimately break through this defense, possibly due to two imbalances: first, the compensatory exhaustion of the antioxidant enzyme system under prolonged stress, and second, the synthesis rate of key defense metabolites (such as Hexanoic acid, pentyl ester) being insufficient to counteract the sustained accumulation of ROS. This finding provides new insights into the host-pathogen interaction mechanisms in postharvest fruit diseases and suggests that exogenous regulation of ROS homeostasis or targeted enhancement of VOC synthesis (such as gene editing of the TPS family) may be potential strategies to improve disease resistance in dragon fruit.

## CRediT authorship contribution statement

**Lili Xie:** Writing – original draft, Data curation, Conceptualization. **Xueli Wang:** Writing – review & editing, Funding acquisition, Conceptualization. **Xiao Ma:** Writing – review & editing, Methodology. **Kaiwei Shen:** Writing – review & editing. **Xuyi Zhang:** Visualization, Methodology. **Xiujun Wang:** Supervision, Formal analysis, Conceptualization.

## Declaration of competing interest

The authors declare that they have no known competing financial interests or personal relationships that could have appeared to influence the work reported in this paper.

## Data Availability

Data will be made available on request.

## Appendix A Supplementary data

Table S1 Detected peak areas of primary metabolites in all groups of dragon fruit. Supplementary material1

Table S2 List of peak areas and classifications of VOCs in dragon fruit. Supplementary material2
